# Supplementary material for: Experimental Identification of Cross-Reacting IgG Hotspots to Predict Existing Immunity Evasion of SARS-CoV-2 Variants by a New Biotechnological Application of Phage Display
Source: Viruses. 2023 Dec 29;16(1):58. doi: 10.3390/v16010058 (PMC10820762; doi:10.3390/v16010058)
Supplement: Supplementary file 1 [file viruses-16-00058-s001.zip › viruses-2744776-supplementary.pdf]

Supplementary material

Supplementary Table S1. Cross-reacting IgG hot-spots in S protein of SARS-CoV-2. Cross-reacting IgG hot spots are regions of protein frequently recognized in many variants by cross-reacting antibodies. Cross-reactions of IgG antibodies were identified by immunoprecipitation of the library containing SARS-CoV-2 variants' oligopeptides (VirScan technology). Immunoprecipitation was conducted with sera from patients hospitalized due to SARS-CoV-2 infection (Alpha or Delta). The fraction of cross-reacting variants of each oligopeptide was calculated from the total number of tested variants. Numbering consistent with the reference sequence (acc. no.: YP_009724390.1). P-values are calculated between measured data and the statistical binomial distribution model assuming random distribution of hot-spots in a protein.

Oligopeptides' position in reference sequence		Number of all tested variants	P-values (exact)		Hot-spot detected	
start	end		alpha	delta	alpha	delta
1	44	18	1.0E+00	1.0E+00	None	None
20	72	70	1.0E+00	1.0E+00	None	None
45	99	173	1.0E+00	1.0E+00	None	None
73	127	124	1.0E+00	1.0E+00	None	None
100	154	74	1.0E+00	1.0E+00	None	None
128	182	29	1.0E+00	1.0E+00	None	None
155	210	96	1.0E+00	1.0E+00	None	None
183	235	137	1.0E+00	1.0E+00	None	None
211	256	165	1.0E+00	1.0E+00	None	None
236	281	106	1.0E+00	1.0E+00	None	None
257	309	115	1.0E+00	1.0E+00	None	None
**282**	**337**	88	**7.2E-29**	**2.3E-23**	**Strong**	**Strong**
310	365	72	1.0E+00	1.0E+00	None	None
338	393	66	1.0E+00	1.0E+00	None	None
366	421	64	9.7E-01	9.9E-01	None	None
**394**	**449**	68	2.0E-04	7.0E-01	**Strong**	None
422	477	103	1.0E+00	9.3E-01	None	None
450	505	163	1.0E+00	9.4E-01	None	None
478	533	128	1.0E+00	1.0E+00	None	None
506	561	92	1.0E+00	9.6E-01	None	None
534	589	111	**5.7E-03**	6.5E-01	Weak	None
**562**	**617**	118	**4.0E-69**	**7.2E-76**	**Strong**	**Strong**
590	645	122	1.0E+00	1.0E+00	None	None
618	672	118	1.0E+00	1.0E+00	None	None
646	696	171	1.0E+00	1.0E+00	None	None
**673**	**724**	200	**6.7E-35**	6.0E-02	**Strong**	None
697	752	127	1.0E+00	1.0E+00	None	None
725	780	96	1.0E+00	1.0E+00	None	None

753	808	110	1.0E+00	9.5E-01	None	None
781	836	144	9.8E-01	7.8E-01	None	None
809	864	135	9.3E-01	4.5E-01	None	None
**837**	**892**	99	1.8E-01	**5.2E-23**	None	**Strong**
865	920	62	1.0E+00	1.0E+00	None	None
893	948	84	1.0E+00	9.8E-01	None	None
921	976	87	9.9E-01	8.0E-02	None	None
**949**	**1004**	45	**3.8E-06**	2.0E-01	**Strong**	None
977	1032	51	1.0E+00	8.8E-01	None	None
1005	1060	61	1.0E+00	8.0E-01	None	None
1033	1088	101	5.2E-01	5.5E-01	None	None
1061	1116	121	1.0E+00	1.0E+00	None	None
1089	1144	122	1.0E+00	1.0E+00	None	None
1117	1172	135	1.0E+00	1.0E+00	None	None
**1145**	**1200**	137	**6.0E-31**	**3.0E-19**	**Strong**	**Strong**
**1173**	**1228**	127	**4.3E-23**	1.7E-01	**Strong**	None
**1201**	**1256**	141	**3.5E-23**	1.0E+00	**Strong**	None
1229	1273	96	1.0E+00	1.0E+00	None	None

Supplementary Table S2. Cross-reacting IgG hot-spots in N protein of SARS-CoV-2. Cross-reacting IgG hot spots are regions of protein frequently recognized in many variants by cross-reacting antibodies. Cross-reactions of IgG antibodies were identified by immunoprecipitation of the library containing SARS-CoV-2 variants' oligopeptides (VirScan technology). Immunoprecipitation was conducted with sera from patients hospitalized due to SARS-CoV-2 infection (Alpha or Delta). The fraction of cross-reacting variants of each oligopeptide was calculated from the total number of tested variants. Numbering consistent with the reference sequence (acc. no.: YP_009724397.2). P-values are calculated between measured data and the statistical binomial distribution model assuming random distribution of hot-spots in a protein.

Oligopeptides position in reference sequence		Number of all tested variants	P-values (exact)		Cross-reacting hot-spots	
start	end		alpha	delta	alpha	delta
1	56	243	1.0E+00	1.0E+00	None	None
29	81	189	1.0E+00	9.3E-01	None	None
**57**	**109**	129	9.9E-01	**5.4E-05**	None	**Strong**
82	137	89	1.0E+00	1.0E+00	None	None
110	165	126	1.0E+00	1.0E+00	None	None
**138**	**193**	186	**1.5E-09**	**8.1E-03**	**Strong**	Weak
**166**	**220**	549	**2.0E-41**	1.0E+00	**Strong**	None
194	245	562	1.0E+00	1.0E+00	None	None
**221**	**273**	184	**2.0E-28**	**5.3E-50**	**Strong**	**Strong**
246	301	123	9.7E-01	9.5E-01	None	None
274	329	103	1.0E+00	1.0E+00	None	None
302	357	112	1.0E+00	1.0E+00	None	None
330	377	141	1.0E+00	1.0E+00	None	None
**358**	**404**	272	**1.2E-04**	**1.0E-11**	**Strong**	**Strong**
**378**	**419**	173	**1.9E-06**	8.7E-03	**Strong**	Weak

Supplementary Table S3. Immunogenicity and cross-reacting IgG hot-spots for M and E protein. Immunogenicity and cross-reactivity were detected by VirScan technology in sera of patients hospitalized due to alpha or delta variant SARS-CoV-2 infection. Below is presented the fraction of patients immunized to a region of reference SARS-CoV-2 protein that recognized the given region significantly more strongly. Also, we show a fraction of protein variants recognized by tested sera for each tested region of proteins. Red shows detected cross-reacting IgG hot spot – regions of protein that in multiple, natural proteins' variants were efficiently bound by antibodies induced by tested sera.

Protein name	Tested oligopeptides' position in ref. sequence [aa no.]	No. of variants tested	Fraction of patients immunized to a region [%]		Fraction of protein variants recognized by tested sera [%]	
	start		alpha	delta	alpha	delta
**M-protein**	1	65	26.7	50.0	6.2	4.6
	29	100	13.3	0.0	**21.0**	3.0
	57	85	0.0	0.0	5.9	0.0
	85	77	0.0	0.0	0.0	0.0
	113	27	26.7	50.0	0.0	0.0
	143	78	0.0	0.0	1.3	0.0
	171	61	20.0	25.0	0.0	0.0
	199	61	0.0	0.0	4.9	1.6
**E-protein**	1	88	0.0	0.0	0.0	0.0
	29	61	0.0	0.0	0.0	0.0
	57	85	0.0	0.0	0.0	0.0

Supplementary Table S4. Immunogenic regions of SARS-CoV-2 protein N. Immunogenic regions efficiently induce specific IgG production in SARS-CoV-2 infected patients. Immunogenic regions were identified by immunoprecipitation of the oligopeptide library representing Alpha and Delta SARS-CoV-2 variants (VirScan technology). Immunoprecipitation was conducted with sera from patients hospitalized due to SARS-CoV-2 infection (Alpha or Delta). Red color represents regions of high and very high immunogenicity ($p<0.05$ and $p<0.001$, respectively). Blue color represents regions of low immunogenicity ($p<0.05$). P-value calculated between experimental data and statistical binomial distribution model assuming random distribution of hot-spot regions in proteins.

Oligopeptides' position in reference sequence		P-values (exact)		Immunogenicity	
start	end	alpha	delta	alpha	delta
**1**	**56**	**1.0E-02**	4.9E-01	**Low**	Medium
29	84	1.4E-01	2.5E-01	Medium	Medium
57	112	3.0E-01	4.9E-01	Medium	Medium
**85**	**140**	**1.1E-03**	**8.8E-03**	**Low**	**Low**
113	168	1.4E-01	7.0E-02	Medium	Medium
141	196	4.9E-01	2.5E-01	Medium	Medium
169	224	3.0E-01	5.1E-01	Medium	Medium
**197**	**252**	5.1E-01	**2.0E-02**	Medium	**High**
**225**	**280**	**9.9E-05**	9.0E-02	**Very High**	Medium
253	308	5.1E-01	4.9E-01	Medium	Medium
281	336	1.4E-01	4.9E-01	Medium	Medium
**309**	**364**	**1.1E-03**	7.0E-02	**Low**	Medium
337	392	5.1E-01	2.3E-01	Medium	Medium
**365**	**419**	**2.9E-07**	**1.7E-02**	**Very High**	**High**

Supplementary Table S5: Immunogenic regions in protein S. Immunogenic regions efficiently induce specific IgG production in SARS-CoV-2 infected patients. Immunogenic regions were identified by immunoprecipitation of the oligopeptide library representing Alpha and Delta SARS-CoV-2 variants (VirScan technology). Immunoprecipitation was conducted with sera from patients hospitalized due to SARS-CoV-2 infection (Alpha or Delta). Red color represents regions of high and very high immunogenicity ($p<0.05$ and $p<0.001$, respectively). Blue color represents regions of low immunogenicity ($p<0.05$). P-value calculated between experimental data and statistical binomial distribution model assuming random distribution of hot-spot regions in proteins.

Oligopeptides' position in reference sequence		P-values (exact)		Immunogenicity	
start	end	alpha	delta	alpha	delta
**1**	**56**	**0.0E+00**	**5.0E-02**	**Low**	**Low**
**29**	**84**	**3.0E-02**	**5.0E-02**	**Low**	**Low**
**57**	**112**	1.0E-01	**5.0E-02**	Medium	**Low**
**85**	**140**	**3.0E-02**	**5.0E-02**	**Low**	**Low**
**113**	**168**	**0.0E+00**	**5.0E-02**	**Low**	**Low**
**141**	**196**	**0.0E+00**	**5.0E-02**	**Low**	**Low**
**169**	**224**	4.7E-01	**1.6E-02**	Medium	**High**
197	252	5.3E-01	2.2E-01	Medium	Medium
**225**	**280**	**0.0E+00**	2.2E-01	**Low**	Medium
253	308	1.0E-01	2.2E-01	Medium	Medium
**281**	**336**	**1.5E-05**	**1.6E-02**	**Very High**	**High**
309	364	4.7E-01	2.2E-01	Medium	Medium
337	392	2.6E-01	2.2E-01	Medium	Medium
365	420	5.3E-01	5.0E-01	Medium	Medium
393	448	1.6E-01	5.0E-01	Medium	Medium
421	476	2.6E-01	5.0E-01	Medium	Medium
**449**	**504**	**3.0E-02**	5.0E-01	**Low**	Medium
**477**	**532**	**3.0E-02**	2.2E-01	**Low**	Medium
505	560	1.6E-01	5.0E-01	Medium	Medium
**533**	**588**	**5.3E-03**	5.0E-01	**High**	Medium
**561**	**616**	**1.0E-03**	7.0E-02	**High**	Medium
**589**	**644**	**3.0E-02**	2.2E-01	**Low**	Medium
617	672	1.0E-01	5.0E-01	Medium	Medium
645	700	1.0E-01	2.2E-01	Medium	Medium
673	728	2.0E-02	2.3E-01	Medium	Medium
**701**	**756**	**0.0E+00**	7.0E-02	**Very High**	Medium
729	784	2.6E-01	2.2E-01	Medium	Medium
**757**	**812**	6.0E-02	**1.6E-02**	Medium	**High**

**785**	**840**	**1.5E-04**	**2.0E-03**	**Very High**	**High**
**813**	**868**	**1.0E-03**	**2.0E-03**	**High**	**High**
841	896	5.3E-01	5.0E-01	Medium	Medium
**869**	**924**	**0.0E+00**	**5.0E-02**	**Low**	**Low**
897	952	5.3E-01	2.3E-01	Medium	Medium
**925**	**980**	**5.3E-03**	**1.6E-02**	**High**	**High**
953	1008	5.3E-01	2.3E-01	Medium	Medium
981	1036	2.6E-01	2.3E-01	Medium	Medium
**1009**	**1064**	**0.0E+00**	5.0E-01	**Low**	Medium
1037	1092	4.7E-01	5.0E-01	Medium	Medium
1065	1120	6.0E-02	5.0E-01	Medium	Medium
1093	1148	1.0E-01	2.2E-01	Medium	Medium
**1121**	**1176**	1.0E-01	**5.0E-02**	Medium	**Low**
**1149**	**1204**	**9.6E-07**	**2.0E-03**	**Very High**	**High**
**1177**	**1232**	**1.5E-05**	**2.0E-03**	**Very High**	**High**
**1205**	**1260**	**3.0E-02**	2.2E-01	**Low**	Medium
**1233**	**1273**	**0.0E+00**	**5.0E-02**	**Low**	**Low**

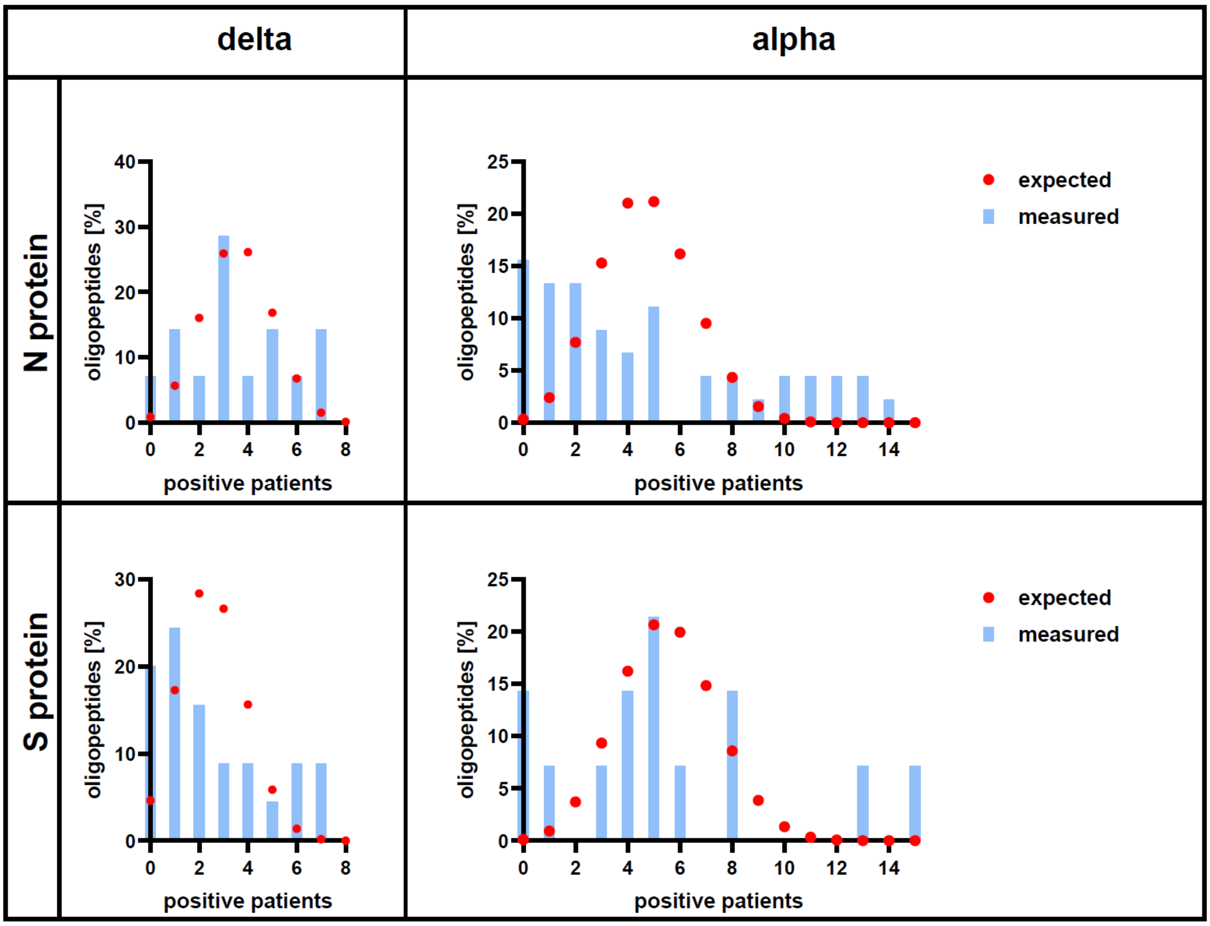

Figure S1. Comparison of binomial distribution model and experimental results of testing proteins' S&N immunogenicity. X-axis shows the number of patients in a tested group (hospitalized for Alpha or Delta variant) whose sera bind the same oligopeptide. Y-axis shows the expected chance of a given number of patients testing positive for the same, given oligopeptide. Blue bars represent measured, experimental frequencies of the given number of positive patients. Red dots represent the chance that measuring this specific number of patients testing positive would occur randomly (binomial distribution model).
